# Factors associated with failure of dog's weight loss programmes

**DOI:** 10.1002/vms3.229

**Published:** 2019-12-26

**Authors:** Mariana Y. H. Porsani, Fábio A. Teixeira, Andressa R. Amaral, Vivian Pedrinelli, Vinícius Vasques, Ariane G. de Oliveira, Thiago H. A. Vendramini, Marcio A. Brunetto

**Affiliations:** ^1^ Veterinary Internal Medicine Department School of Veterinary Medicine and Animal Science University of São Paulo – USP São Paulo Brazil; ^2^ Nutrition and Animal Production Department School of Veterinary Medicine and Animal Science University of São Paulo – USP São Paulo Brazil

**Keywords:** canine, compliance, diet, nutrition, obesity

## Abstract

**Background:**

Canine obesity is the cause of several health issues, and may predispose other diseases, such as orthopaedic disorders, endocrinopathies, metabolic abnormalities and cardiorespiratory disease which can lead to a decreased quality of life and reduced lifespan. Dog are considered overweight when their body weight is ≥15% above their ideal body weight, and as obese when their body weight exceeds 30% of optimal. Prevalence of canine obesity is estimated to be around 5%–20%, and up to 30%–40% when all overweight dogs are considered. Treatment is based on weight loss programmes, focused on caloric restriction associated with exercise. However, success rate of treatment is low.

**Objectives:**

The aim of this study was to investigate the reasons for weight loss failure of obese dogs during treatment with low‐calorie diets.

**Methods:**

Records of obese dogs undergoing weight loss programmes between May 2014 and May 2017, assessed by a team specialized in veterinary nutrition, were retrospectively evaluated. Dogs were classified according to weekly weight loss rate (WWLR) (<1%, between 1% and 2%, and more than 2%) and owner compliance with a dietary prescription and physical activity recommendations.

**Results:**

The mean WWLR was not satisfactory (<1%) in 64.4% (*n* = 47/73) of dogs. Regarding owner compliance with the dietary prescription, 44.7% (*n* = 21/47) of owners did not follow diet prescriptions and physical activity recommendations for their dogs. There was a significant association (*p* = .01) between compliance of owners and satisfactory weight loss rate; however, there was no association between weight loss success, diet composition sex, reproductive status, age of the dogs and their physical activity (*p* ≥ .05).

**Conclusions:**

Non‐compliance represents a great challenge in the treatment of canine obesity, and may be of more importance than other aspects.

## Graphical abstract

Association between features of 73 obese dogs undergoing a weight loss program and weekly weight loss rate.

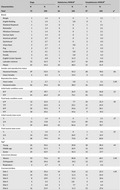

## 1 INTRODUCTION

The number of overweight dogs has increased significantly over the years, and it is estimated that about 30%–40% of the canine population is in overweight, ≥15% above their ideal body weight, and 5%–20% have a body weight which exceeds 30% of their optimal and are considered obese (Alonso et al., 2017; Courcier, Thomson, Mellor, & Yam, 2010; Mao, Xia, Chen, & Yu, 2013).

Excess body fat is a consequence of prolonged imbalance between food intake and energy expenditure (Courcier et al., 2010). Chronic positive energy balance may be related to genetic, environmental and human behavioural and sociocultural factors (Alonso et al., 2017; German, 2015). In dogs, neutering and increasing age are associated with a reduction in metabolic rate and changes in body composition because of changes in eating behaviour and voluntary physical activity, predisposing these dogs to obesity (Courcier et al., 2010; Kawauchi et al., 2017; Lund, Armstrong, Kirk, & Klausner, 2006; McGreevy et al., 2005; Robertson, 2003).

Excess body fat is also related to endocrine and inflammatory profile changes in dogs, and may predispose to organic alterations that culminate in decreased quality of life and reduced lifespan (Alonso et al., 2017; German et al., 2012; Huck et al., 2009; Kealy et al., 2002; Vitger, Stallknecht, Nielsen, & Bjornvad, 2016). Consequences of obesity include insulin resistance, modification of adipokine patterns, lipid metabolism changes, ectopic fat accumulation, pulmonary and cardiac diseases, non‐allergic dermatitis, pancreatitis, neoplasia and nephropathies (Brunetto et al., 2011; Chandler, 2016; Clark & Hoenig, 2016; German, Ryan, German, Wood, & Trayhurn, 2010; Michel, 2012; Toll, Yamka, Schoenherr, & Hand, 2010). Furthermore, obesity can cause joint overload, interfere with the immune system and alter cytokine production which can reach different tissues and intensify the inflammatory response (Chandler, 2016; Huck et al., 2009; Marshall, Bockstahler, Hulse, & Carmichael, 2009; Michel, 2012; Toll et al., 2010).

Thus, the implementation of an adequate weight loss programme must be established by modification of eating habits and increasing physical activity (Flanagan et al., 2017; Robertson, 2003; Vitger et al., 2016). Providing balanced and complete commercial diets with lower energy density and higher protein and fibre concentrations favours adequate satiety and prevents malnutrition and excessive loss of lean body mass (LBM) during the weight loss programme (Linder et al., 2012).

Although energy restriction and increased physical activity are important strategies, owner compliance with the protocol plays a key role in the success of the weight loss programme (Brooks et al., 2014; Saker & Remillard, 2005). Saker and Remillard (2005) performed a study in which they compared compliance of owners according to feeding practices and frequency of reassessments and concluded that frequency of reassessments contributed to the success of weight loss programmes.

Many owners consider their pets as family members and think that food restriction may lead to possible distress of the animal. As a consequence, they may not comply with the programme of weight loss because they do not want to stop giving snacks or control the quantity of food (Charles & Davies, 2008; Yaissle, Holloway, & Buffington, 2004). This is observed in obese children, as their parents tend not to follow recommendations for weight loss and this impairs the success of treatment (Flanagan et al., 2017; German, 2015; Linder et al., 2012; Pretlow & Corbee, 2016).

In view of the difficulties encountered in the management of weight loss programmes in dogs, the objective of this study was to investigate the possible reasons associated with success and failure regarding current recommendation strategies for canine obesity.

## 2 MATERIALS AND METHODS

Medical records of obese dogs that were assessed by a specialized veterinary nutrition team of a teaching hospital, between May 2014 and May 2017, were retrospectively evaluated. Inclusion criteria were dogs with a body condition score (BCS) ≥8, on a 9‐point scale (Laflamme, 1997), that were undergoing a weight loss programme and that returned for reassessments at least four times. Dogs with comorbidities able to impair or contribute to weight loss, as well as dogs that received drugs with potential appetite stimulating properties, such as anticonvulsants or steroids, were excluded. Dogs that, for whatever reason, did not consume extruded therapeutic diets for weight loss were not included. The weight loss protocol used is as described by Brunetto et al. (2011) and Brooks et al. (2014), in which a target body weight of 80% the initial weight is established, and daily weight loss energy requirement (DWLER) calculated according to the equation: DWLER (kcal) = 70 × target body weight (kg)^0.75^. In every assessment, dogs were weighed and BCS (Laflamme, 1997) and muscle mass score (Michel, Anderson, Cupp, & Laflamme, 2011) were determined. Weekly weight loss rate (WWLR) was calculated as a percentage according to previous assessments of body weight and number of weeks between reassessments. WWLRs were classified was satisfactory (1%–2%) and unsatisfactory (<1%). In each reassessment, caloric intake was readjusted if necessary, with a 10% increase or reduction if the WWLR was higher than 2% or lower than 1%, respectively.

Information regarding the first four visits was considered. In the first one (T0 = dog initial assessment), a review was carried out with information about the dog, characteristics of nutritional management and information about physical activity practice were collected from the owner. After the beginning of the weight loss programme, dogs assessment at three time points (T1, T2 and T3). At these time points, the owners gave information about dog's general state of health of dogs, how they quantified the amount of food offered, which dog food they bought (diet name and manufacturer). Also, the owners reported whether the dog ate any unprescribed snacks and whether they practice of physical activity (dogs who walked at least 20 min twice a week with their owners). Besides, the veterinarian team evaluated the BCS classification, weighed the dogs and calculated the WWLR, and if necessary, recommended (re)adjustment of caloric intake and improvement of physical activity.

Owner compliance with the prescription was considered satisfactory when owners mentioned weighing the prescribed amount of food, not providing any extra food or snacks, and performing physical activity according to the recommendation. Recommended a physical activity practice was that owners walked their dogs at least twice a week, for at least 20 min. Play with other animals was not considered as activity in this study.

Data were evaluated using descriptive statistics, expressed in percentages. To evaluate associations between weight loss rate and categorical variables such as dog sex, neutering status, age range; diet prescribed, owner compliance and physical activity, a chi‐square test was used with a significance a level of 5%. For quantitative variables such as days between reassessments and WWLR, data were tested for normality with a Shapiro–Wilk test, and normally distributed variables were analysed with a Student's *t* test, and variables with non‐normal distribution were analysed with a Wilcoxon test. Analyses were performed with GraphPad Prism 6 software (GraphPad Software Inc).

## 3 RESULTS

Within the 3‐year period, 322 medical records were obtained for dogs with BCS ≥8. In all, 91 dogs were excluded because of comorbidities or due to use of medication that could influence weight loss. Another 92 dogs were excluded because they consumed diets that were not specific therapeutic diets for treatment of obese dogs, and 66 dogs were excluded for missing at least three scheduled appointments. Therefore, 73 dogs were included in this study. Characteristics of these dogs are described in Table 1.

**Table 1:** Association between features of 73 obese dogs undergoing a weight loss programme and WWLR

Characteristics	Dogs		Satisfactory WWLR^a^		Unsatisfactory WWLR^b^		*p* *
	*N*	%	*N*	%	*N*	%	
Total	73	100	26	100	47	100	
Breed							
Beagle	1	1.4	0	0	1	2.1	—
English Bulldog	1	1.4	1	3.8	0	0	
Shetland Shepherd	1	1.4	0	0	1	2.1	
Rottweiler	1	1.4	0	0	1	2.1	
Miniature Schnauzer	1	1.4	0	0	1	2.1	
German Spitz	1	1.4	0	0	1	2.1	
American pit bull	2	2.7	0	0	2	4.3	
Dachshund	2	2.7	0	0	2	4.3	
Lhasa Apso	2	2.7	1	3.8	1	2.1	
Pug	2	2.7	0	0	2	4.3	
Golden Retriever	3	4.1	1	3.8	2	4.3	
Poodle	3	4.1	1	3.8	2	4.3	
English Cocker Spaniel	5	6.8	3	11.5	2	4.3	
Labrador retriever	23	31.5	9	34.7	14	29.8	
Mixed	25	34.2	10	38.5	15	31.9	
Gender and reproductive status							
Neutered females	39	53.4	11	42.3	28	59.6	.06
Intact females	6	8.2	4	15.4	2	4.3	
Reproductive status							
Intact males	2	2.7	1	3.8	1	2.1	1.00
Neutered males	26	35.6	10	38.5	16	34.0	
Initial body condition score							
8/9	29	39.7	7	26.9	22	46.8	.13
9/9	44	60.3	19	73.1	25	53.1	
Final body condition score							
6/9	12	16.4	2	7.7	10	21.3	.42
7/9	17	23.3	6	23.1	11	23.4	
8/9	26	35.6	9	34.6	17	36.1	
9/9	18	24.6	9	34.6	9	19.2	
Initial muscle mass score							
¼	1	1.4	0	0	1	2.1	.27
2/4	13	17.8	4	15.4	09	19.1	
¾	59	80.8	22	84.6	37	78.7	
Final muscle mass score							
¼	1	1.4	0	0	1	2.1	.31
2/4	14	19.2	7	26.9	7	14.9	
¾	58	79.4	19	73.0	39	83.0	
Age^c^							
Young	26	35.6	8	30.8	18	38.3	.44
Middle age	23	31.5	7	26.9	16	34.0	
Senior	24	32.9	11	42.3	13	27.7	
Concurrent disease							
Absent	53	72.6	21	80.8	32	68.1	1.00
Orthopaedic	18	24.6	05	19.2	13	27.7	
Respiratory	2	2.7	0	0	2	4.2	
Commercial diets							
Diet 1	26	35.6	14	53.8	12	25.5	≥.05
Diet 2	14	19.2	3	11.5	11	23.4	
Diet 3	8	10.9	3	11.5	5	10.6	
Diet 4	4	5.5	1	3,8	3	6.4	
Diet 5	5	6.8	2	7.7	3	6.4	
Diet 6	16	21.9	3	11.5	13	27.6	

Abbreviation: WWLR, weekly weight loss rate.

a Mean WWLR ≥1%.

b Mean WWLR <1%.

c Criteria for age class by size (Hosgood & Scholl, 1998).

* Values of chi‐square test.

Dogs in the study consumed six different therapeutic diets for obesity treatment. However, the diets in their formulation (e.g. moisture, fat, fibre, protein and nitrogen‐free extract content, according to label information; Table 2). All diets were designed to be complete and balanced for all essential nutrients even when fed for negative energy balance (e.g. less than maintenance requirements).

**Table 2:** Chemical composition of commercial extruded weight loss diets consumed by 73 obese dogs enrolled in a study to investigate factors affecting the success of a weight loss programme

	Energy (kcal/kg*)	Protein (g/1000 kcal)*	Fat (g/1000 kcal)*	Crude fibre (g/1000 kcal)*	Ash (g/1000 kcal)*	NFE^a^ (g/1000 kcal)*
Diet 1	3,161	10.7	3.5	1.4	2.2	11.2
Diet 2	2,979	11.9	2.6	5.0	2.5	11.4
Diet 3	2,870	9.7	2.6	6.3	2.2	13.9
Diet 4	3,118	10.2	2.5	3.3	2.9	13.0
Diet 5	2,850	10.5	2.8	4.2	2.9	14.6
Diet 6	2,990	8.7	2.8	4.3	2.5	13.9

a NFE = nitrogen‐free extract, calculated according to the equation: 100 − [moisture (%) + protein (%) + fat (%) + ash (%) + crude fibre (%)].

* According to the information provided by manufactures on the labels of the products.

Considering all reassessments, 35.6% (*n* = 26/73) of dogs achieved satisfactory WWLR (mean 1.27 ± 0.25%) and 64.4% (*n* = 47/73) had an unsatisfactory WWLR (mean 0.55 ± 0.28%) (*p* < .0001). Median time between assessments was 19.6 ± 7.1 days for the satisfactory WWLR group and 33.7 ± 7.1 days for the unsatisfactory WWLR group (*p* = .026).

Of dogs that did not lose weight at a satisfactory WWLR, 29.8% (*n* = 14/47) did not change BCS, 38.3% (*n* = 18/47) reduced one point on the BCS scale, 23.4% (*n* = 11/47) reduced two points, 6.4% (*n* = 3/47) reduced three points and 2.1% (*n* = 1/47) reduced five points on the BCS scale. Of dogs that lost weight at a satisfactory WWLR, 42.3% (*n* = 11/26) did not change BCS, 38.5% (*n* = 10/26) reduced one point on the BCS scale, 19.2% (*n* = 5/26) reduced two points and 19.2% (*n* = 5/26) reduced three points on the BCS scale.

Age range (*p* = .44) and diet (*p* > .05) were not associated with WWLR, as well as sex (*p* = .62) and neutered dogs independent of sex (*p* = .12). When sex and neutering status were both considered as variables, there was still no association with WWLR (*p* > .05). As there was an uneven breed distribution of dogs, association for this variable was not tested.

Adjustments in the caloric amounts prescribed were made at different time points (T1, T2 and T3). Dogs that did not obtain satisfactory weight loss had readjustments made at T1 (48.9%; *n* = 23/47), T2 (53.1%; *n* = 25/47) and T3 (42.5%; *n* = 20/47). Of the dogs that achieved satisfactory WWLR, readjustments were made in 19.2% (*n* = 5/26) of dogs between T1 and T2 reassessments and 23.0% (*n* = 6/26) dogs between T2 and T3.

All owners (*n* = 26/26) of dogs who had satisfactory weight loss complied with the recommendations made by the veterinary team in contrast to the owners of dogs with unsatisfactory weight loss rates where the majority 85.5% (*n* = 40/7) did not follow the recommendations. There was an association between compliance with feeding recommendations and satisfactory weekly weight loss (*p* = .03). Although physical activity was recommended to all dogs, this practice was performed only by 53.4% (*n* = 39/73) of the owners. Among those, 57.7% (*n* = 15/26) had satisfactory weekly weight loss and 51.1% (*n* = 24/47) did not and, therefore, no association between the two variables was found (*p* = .58). An association between adherence to the weight loss programme as a whole (amount of food and physical activity prescribed) and weight loss rate was observed (*p* = .01).

## 4 DISCUSSION

Weight loss programmes based on caloric restriction, a weight loss prescription diet and physical activity practice should be supervised so that the WWLR is adequate and to ensure a healthy process (Brooks et al., 2014; Saker & Remillard, 2005). Despite being a retrospective study, all dogs were subjected to the same weight loss protocol. Different diets were employed, but all were indicated for obesity treatment and were not associated with the programme success.

A WWLR between 1% and 2% is a suggested objective in weight loss programmes for dogs, based on experimental studies with dog colonies (Brooks et al., 2014; Floerchinger et al., 2017, 2015). Although 64.4% of dogs in this study did not achieve a weight loss rates between these values, only 29.8% of dogs in this category maintained BCS, which suggests that even if the WWLR is lower than 1%, dogs still lost weight. This result is similar to that observed by Saker and Remillard (2005), who observed a mean WWLR of 0.75%.

Monitoring the dogs during the period of weight loss is essential for the success of the programme and readjustments in the caloric amount should be made when the weight loss rate is not satisfactory (Flanagan et al., 2017; Saker & Remillard, 2005; Yaissle et al., 2004). In the present study, these readjustments allowed the improvement of the subsequent WWLRs.

Dogs‐related factors, such as age and reproductive status, may influence the success of the weight loss programme or even be associated with weight gain (Courcier et al., 2010; Kawauchi et al., 2017; Vitger et al., 2016; Zoran, 2010). These factors were related in a study conducted by Flanagan et al. (2017) in which the authors observed higher weight loss in intact animals. This may be due to a reduction in metabolism and energy requirements after neutering (Robertson, 2003). However, in the present study, dog sex and neutering status were not associated with WWLR, and this may be due the majority of the study population being female and neutered.

Some studies have pointed out that changes in the attitude of the owners with regard to implementation of physical activity and energy expenditure increase favour the maintenance of LBM during weight loss (Brooks et al., 2014; Chauvet, Laclair, Elliott, & German, 2011; Vitger et al., 2016; Yaissle et al., 2004). In the present study, most of the owners preferred to walk with their dogs while performing other activities such as run or swimming. Rather than running or perform higher impact's activity and although this attitude did not improve the weight loss rate, LBM was maintained in all dogs throughout the evaluation period. However, the ideal frequency and type of exercise necessary to promote weight loss and LBM were maintain still not defined yet (Chauvet et al., 2011; Vitger et al., 2016).

The success of any weight loss programmes depends on compliance of the owner with the treatment protocol, and therefore it is of utmost importance that veterinarians assess the accuracy with which their nutritional prescription is followed (Flanagan et al., 2017; Saker & Remillard, 2005). However, several studies observed that owners tend to modify nutritional prescription without the veterinarian's consent (Bland, Guthrie‐Jones, Taylor, & Hill, 2009; German, Holden, Gernon, & Morris, 2011; Roudebush, Schoenherr, & Delaney, 2008). Some owners of dogs who did not achieve a satisfactory weight loss rate provided non‐prescribed food and did not follow the recommendations for exercise, which negatively affected the outcome of the treatment for canine obesity.

Studies in humans observed that non‐compliance with nutritional prescriptions occurs in up to 50% of diabetic and chronic kidney disease patients (Pontieri & Bachion, 2010). This high incidence of non‐compliance was attributed to cultural, emotional, ethnic and economical factors, and also to difficulty in comprehending the prescription (Emadian, England, & Thompson,^2017^; Pierri, Zago, & Mendes,^2015^). Social and behavioral factors along with a low levels of education also contribute to non‐compliance. Furthermore, the severity of the clinical condition may interfere with compliance, since patients with fewer symptoms tend not follow to through with nutritional prescriptions (Pontieri & Bachion, 2010). In the present study, most dogs did not present comorbidities, which may have caused higher non‐compliance rates.

Good communication is important for adherence to diet and physical activity practice recommendations (German, 2015; Michel et al., 2008). The possibility that the owners did not understand how to follow the prescription may explain the high rate of non‐compliance; however, the design of this study did not allow this type of analysis.

As a limitation, the present study is retrospective. Therefore, as the population is not randomized, there is an uneven distribution between dog breeds, sex and neutering status in the study population, which may have affected results.

Based on this study, it can be concluded that among the factors investigated, the main point related to adequate WWLR is compliance with treatment recommendations, as non‐compliance with the exercise and feeding recommendations were the main factors responsible for unsatisfactory results. This demonstrates that the good communication to the owner about education, the consequences of obesity and the importance of following recommendations for the treatment of obesity should be made more emphatically to owners. It is ensure that owners understand the recommendations.

## CONFLICT OF INTEREST

The authors declare that they have no conflict of interest.

## ETHICAL APPROVAL

The authors confirm that the ethical policies of the journal, as noted on the journal's author guidelines page, have been adhered to and the appropriate ethical review committee approval has been received. The US National Research Council's guidelines for the Care and Use of Laboratory Animals were followed.
